# Pre-Immunotherapy Contrast-Enhanced CT Texture-Based Classification: A Useful Approach to Non-Small Cell Lung Cancer Immunotherapy Efficacy Prediction

**DOI:** 10.3389/fonc.2021.591106

**Published:** 2021-04-23

**Authors:** Leilei Shen, Hongchao Fu, Guangyu Tao, Xuemei Liu, Zheng Yuan, Xiaodan Ye

**Affiliations:** ^1^Department of Radiology, Shanghai Chest Hospital, Shanghai JiaoTong University, Shanghai, China; ^2^Department of Radiology, Huadong Hospital, Fudan University, Shanghai, China

**Keywords:** texture, immunotherapy, radiomics, response prediction, non-small cell lung cancer

## Abstract

**Objective:** To investigate the utility of the pre-immunotherapy contrast-enhanced CT-based texture classification in predicting response to non-small cell lung cancer (NSCLC) immunotherapy treatment.

**Methods:** Sixty-three patients with 72 lesions who received immunotherapy were enrolled in this study. We extracted textures including histogram, absolute gradient, run-length matrix, gray-level co-occurrence matrix, autoregressive model, and wavelet transform from pre-immunotherapy contrast-enhanced CT by using Mazda software. Three different methods, namely, Fisher coefficient, mutual information measure (MI), and minimization of classification error probability combined average correlation coefficients (POE + ACC), were performed to select 10 optimal texture feature sets, respectively. The patients were divided into non-progressive disease (non-PD) and progressive disease (PD) groups. *t*-test or Mann–Whitney *U*-test was performed to test the differences in each texture feature set between the above two groups. Each texture feature set was analyzed by principal component analysis (PCA), linear discriminant analysis (LDA), and non-linear discriminant analysis (NDA). The area under the curve (AUC) was used to quantify the predictive accuracy of the above three analysis models for each texture feature set, and the sensitivity, specificity, accuracy, positive predictive value (PPV), and negative predictive value (NPV) were also calculated, respectively.

**Results:** Among the three texture feature sets, the texture parameter differences of kurtosis (2.12 ± 3.92 vs. 0.78 ± 1.10, *p* = 0.047), “S(2,2)SumEntrp” (1.14 ± 0.31 vs. 1.24 ± 0.12, *p* = 0.036), and “S(1,0)SumEntrp” (1.18 ± 0.27 vs. 1.28 ± 0.11, *p* = 0.046) between the non-PD and PD group were statistically significant (all *p* < 0.05). The classification result of texture feature set selected by POE + ACC and analyzed by NDA was identified as the best model (AUC = 0.812, 95% CI: 0.706–0.919) with a sensitivity, specificity, accuracy, PPV, and NPV of 88.2, 76.3, 81.9, 76.9, and 87.9%, respectively.

**Conclusion:** Pre-immunotherapy contrast-enhanced CT-based texture provides a new method for clinical evaluation of the NSCLC immunotherapy efficacy prediction.

## Introduction

In recent years, with the development of tumor immunology research, many breakthroughs have been made in tumor immunotherapy. Some immunotherapy has significantly prolonged the survival of tumor patients and improved the quality of life (1, 2). In the second-line treatment of non-small cell lung cancer (NSCLC), immune checkpoint inhibitors have made progress. From Checkmate-017 and Checkmate-057 studies to KEYNOTE-010 and OAK studies, they have gradually established the programmed death-1/programmed death ligand-1 (PD-1/PD-L1) inhibitors as standard treatment for advanced NSCLC after chemotherapy failure (3, 4). Although solid tumor immunotherapy is currently being widely carried out clinically and has achieved some exciting results, there are still many unresolved problems, such as the lack of effective methods for immunotherapy to find individual tumor-specific targets (5). Among these problems, how to accurately evaluate the efficacy of immunotherapy at an early stage is still a difficult problem for clinicians when making clinical treatment decisions. Recently, with the development of medical image informatics, extraction of image features and analyzing clinical information have gradually attracted the attention of medical experts. In particular, the research results of radiomics for the evaluation of efficacy (6) and prognosis (7) have a potentially great value for guiding and optimizing clinical decisions and achieving individualized and precise treatment of lung cancer.

In our study, we extracted and analyzed the texture features of enhanced CT images of NSCLC before immunotherapy to evaluate its feasibility and clinical application value for predicting the efficacy of tumor immunotherapy.

## Materials and Methods

### Subjects

Our Institutional Review Board approved this retrospective study and waived the need for informed consent from the patients. From January 2018 to February 2019, patients of our hospital with advanced-stage NSCLC receiving PD-1/PD-L1 inhibitor nivolumab immunotherapy were selected in this study. Inclusion criteria are as follows: (1) patients underwent contrast-enhanced CT in our hospital within 1 week before receiving tumor immunotherapy; (2) with measurable lesions for the evaluation of efficacy; and (3) at least one follow-up data were used to evaluate the efficacy.

### CT Screening

CT scans were obtained with a 128-detector row scanner (Brilliance, Philips, Cleveland, OH, USA) using the helical technique at the end of inspiration during one breath hold. The scanning parameters of routine CT were as follows: pitch, 1.0; matrix, 1,024 × 1,024; FOV, 300 mm; 120 kVp and 200 mA. After non-enhanced CT scanning, a double-cylinder high-pressure syringe pump was used to inject 2 ml/kg BW of iodine contrast agent (Iophorol 320 mg I/ml) into the elbow vein, with an 18-gauge needle, followed by 20 ml of normal saline at a flow rate of 3 ml/s. Enhanced CT scans were acquired 25 and 75 s after drug infusion, respectively. The scanning range covered the entire area from the apex to the base of the lung with the patient lying supine, which included adrenal glands on both sides. When a lesion was found, an HRCT target scan between arterial phase and delay-enhanced scan followed with the following parameters: pitch, 1.0; section thickness and interval, 1.0 and 1.0 mm; matrix, 1,024 × 1,024; FOV, 150 mm; 120 kVp and 200 mA. The images of the contrast-enhanced CT lesions (HRCT target scans) were stored as Dicom for image texture feature extraction.

#### Image Segmentation and Feature Extraction

All raw thin-slice DICOM format images of the contrast-enhanced CT lesions (HRCT target scans) were transferred to Mazda software (The Technical University of Lodz, Institute of Electronics, http: //www.eletel. P.lodz.pl/mazda/). Tumors were segmented by two radiologists with different experience in thoracic oncological imaging (5 and 15 years). The primary radiologist selected the largest section of the lesion, manually drawing the ROI diagram, and then the experienced senior radiologist confirmed the ROI setting, taking the lead when the two radiologists disagreed. The specific methods and steps are as follows:

1. ROI is drawn on the enhanced CT image of the median window (width, 360 HU; level, 60 HU) at the central level of the cross-section of each target lesion. The two radiologists were mainly responsible for delineating the boundary of each primary tumor manually layer by layer, which required to include all lesions as much as possible.
2. After ROI, the texture parameters of the images of the lesions within the range shown by the ROI are calculated by the Mazda software;
3. Feature extraction

Since there are many texture feature parameters extracted by the Mazda software, we chose three methods for screening feature texture parameter with clinical interpretation, namely: Fisher coefficient, mutual information (MI), and classification error probability combined average correlation coefficients (POE + ACC). We selected all screen 10 characteristic texture parameters from the above three methods.

#### Tumor Immunotherapy and Evaluation Methods

All patients received a treatment of nivolumab (OPDIVO, Bristol-Myers Squibb Company), 240 mg, once every 2 weeks. Tumor assessments were performed every 6–8 weeks by contrast-enhanced computed tomography (CT) scan after the start of treatment. We only evaluate target lesions in the mediastinal window, including primary lesions or metastases, while we do not calculate changes in lesions outside the lung parenchyma such as lymph nodes. According to RECIST 1.1 standard (8), the longest diameters of target lesions were recorded by two chest radiologists, centrally reviewed all consecutive CT scans independently. When the results are different, another oncologist joined to discuss the decision. Complete response (CR) was defined as the disappearance of all lesions. Partial response (PR) was more than 30% decrease in the sum of the longest diameters of the target lesions. Suspicion of progression was recorded as immune unconfirmed progressive disease (iUPD) according to the iRECIST guideline (9). Oncologists judged whether to continue treatment integrately based on the patient's tumor type, disease stage, and clinical situation. Another evaluation of contrast-enhanced CT was preformed 4–6 weeks later to confirm the true progressive disease (iCPD). Progressive disease (PD) was defined as a more than 20% increase in the sum of the longest diameters of the target lesions. A patient who could not be classified as having either PR or PD was diagnosed as having stable disease (SD). Patients were divided into the non-progressive group (including CR, PR, and SD) and the progressive group (PD) on the basis of the follow-up CT scan date after the first cycle immunotherapy.

### Statistical Analysis

*t*-test (categorical data) or Chi-square test (enumeration data) was performed to compare the differences of the clinical characteristics between non-PD and PD patients. *t*-test (normal distribution data) or Mann–Whitney *U* (non-normal distribution data) was performed to compare the radiomics texture features extracted by Fisher coefficient, mutual information measure (MI), and minimization of classification error probability combined average correlation coefficients (POE + ACC) between the non-progressive disease (non-PD) group and the progressive disease (PD) group. According to the selected texture features, the B11 statistical software module included in the Mazda software package is used to classify the predictive effect of tumor immunotherapy target lesions. Classification methods include linear discriminant analysis (LDA), non-linear discriminant analysis (NDA), and principal component analysis (PCA). Based on the texture features of the pre-immunotherapy contrast-enhanced CT, we calculated the sensitivity, specificity, accuracy, positive predictive value, and negative predictive value of each classification method by SPSS 22.0 software, to predict the efficacy of NSCLC immunotherapy and calculate the area under the curve (AUC) to compare the effectiveness of various classification methods to predict the efficacy.

## Result

A total of 63 NSCLC patients (51 males and 12 females, with an average age of 61.2 years and a range of 40–79 years) were analyzed. The clinical characteristics of the patients are shown in Table 1. There were 72 lesions, in which 39 were non-progressive lesions (including 12 PR and 27 SD) and 33 were progressive lesions, divided into two groups based on the evaluation of immune efficacy. When there were multiple target lesions in the same patient, the efficacy was consistent.

**Table 1:** Clinical characteristics of patients.

		**Non-PD group**	**PD group**	***p*-value**
		**(*n* = 39)**	**(*n* = 33)**	
Patients		34	29	
Age		62.0	60.7	0.387^*^
Sex				0.342
	Male	29	22	
	Female	5	7	
Smoking status				0.176
	Current smoker	26	16	
	Never smoker	6	11	
	Former smoker	2	2	
Histology				0.758
	Adenocarinoma	21	19	
	Squamous cell carcinoma	13	10	
Stage				0.066
	III	13	5	
	IV	21	24	
Previous therapy				0.805
	Treatment naïve	0	1	
	Exclusively chemotherapy/TKI	21	16 (1 TKI)	
	Chemotherapy + Radiochemotherapy	8	8	
	Chemotherapy + Radiochemotherapy + Surgery	2	1 (surgery of brain metastasis)	
	Chemotherapy + Surgery	3	3	
Target lesion	Total	39	33	0.126
	Right upper lobe	12	14	
	Right middle lobe	0	0	
	Right lower lobe	9	4	
	Left upper lobe	10	4	
	Left lower lobe	8	8	
	Two lobes or more	0	3	

* *p-value is obtained by the t-test; otherwise, p-value is obtained by Chi-square test*. *Non-PD group, non-progressive group; PD group, progressive group*.

### Difference of the Feature Textures Extracted by the Three Methods Between the Non-progressive Group and the Progressive Group

**Figure 1:**
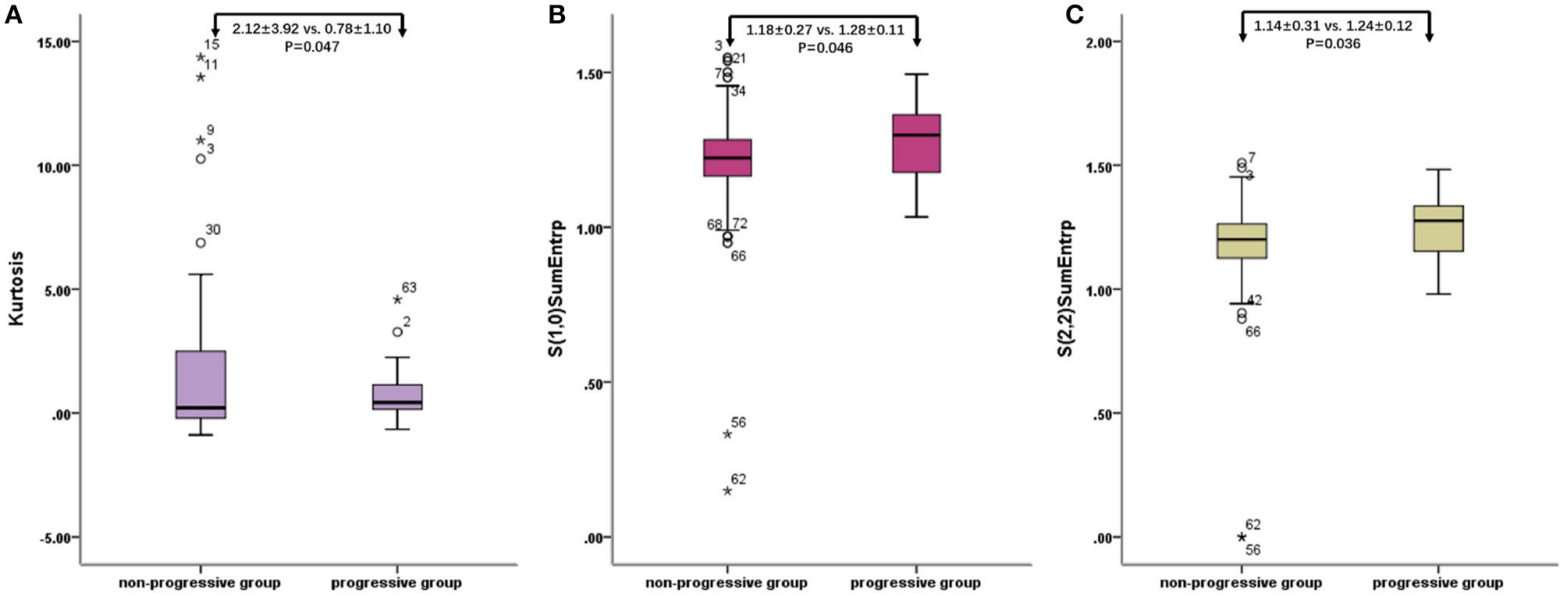
Radiomic features of baseline contrast-enhanced CT: box plot of Kurtosis **(A)**, “S(2,2)SumEntrp” **(B)**, and “S(1,0)SumEntrp” **(C)**. o stands for outlier.

**Figure 2:**
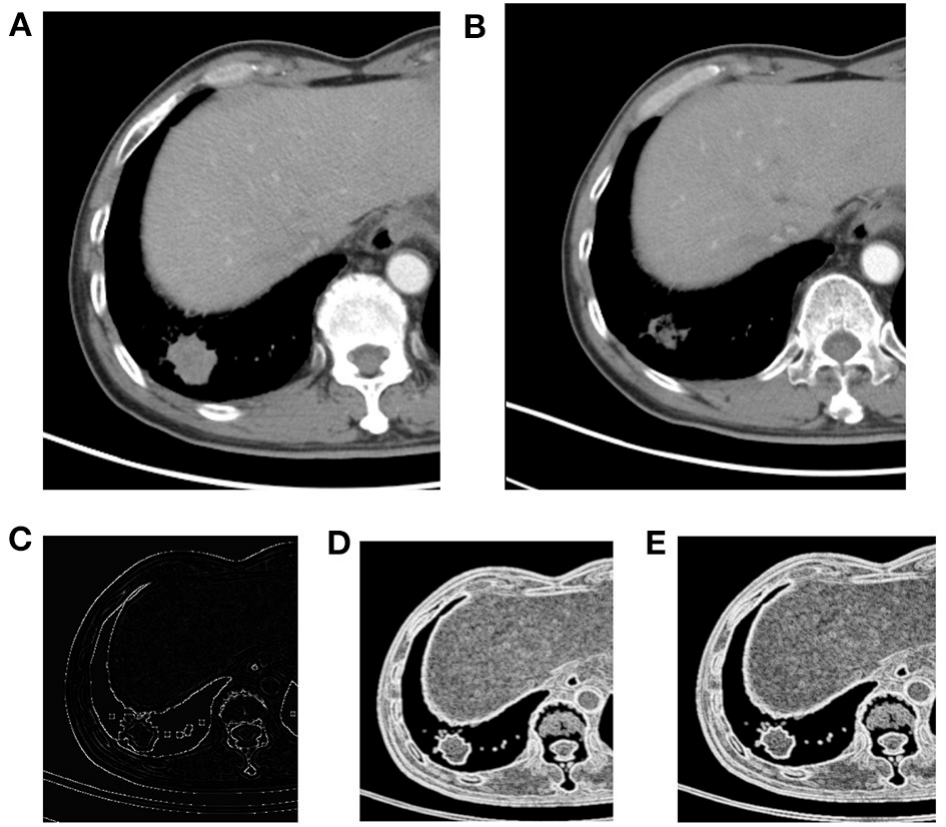
Right lower lobe nodule, NSCLC. **(A)** Pre-treatment contrast-enhanced; **(B)** contrast-enhanced CT 6 weeks later after treatment, the efficacy evaluation was partial response (PR); **(C)** kurtosis; **(D)** S(1,0) SumEntrp map; **(E)** S(2,2) SumEntrp map.

**Figure 3:**
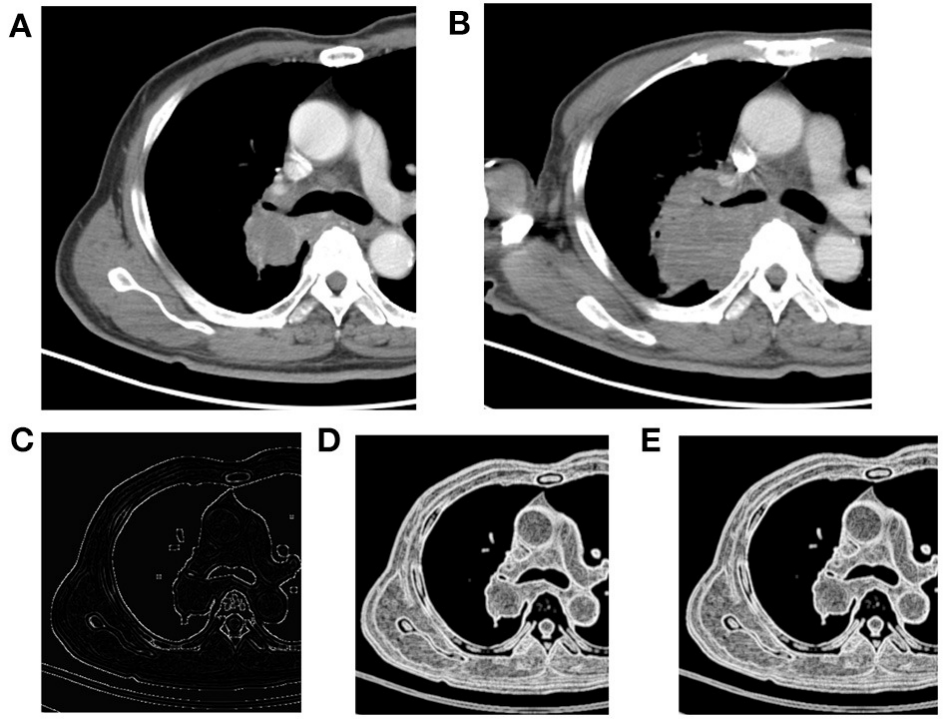
Right upper lobe mass, NSCLC. **(A)** Pre-treatment contrast-enhanced CT; **(B)** contrast-enhanced CT 8 weeks later after treatment, the efficacy evaluation was progression (PD); **(C)** kurtosis map; **(D)** S(1,0) SumEntrp map; **(E)** S(2,2) SumEntrp map.

The characteristic texture parameters extracted by Fisher coefficient, MI, and the POE + ACC method are shown in Table 2. Three radiomics features that were statistically significant between the non-progressive group and the progressive group (Figure 1) were as follows: kurtosis (2.12 ± 3.92 vs. 0.78 ± 1.10, *p* = 0.047), “S(2,2)SumEntrp” (1.14 ± 0.31 vs. 1.24 ± 0.12, *p* = 0.036), and “S(1,0)SumEntrp” (1.18 ± 0.27 vs. 1.28 ± 0.11, *p* = 0.046), among which kurtosis is the parameter of grayscale histogram. The values of “S(2,2)SumEntrp” and “S(1,0)SumEntrp” were larger in the progress group than in the non-progress group. “S(2,2)SumEntrp” and “S(1,0)SumEntrp” are the parameters and entropy of the gray-level co-occurrence matrix. The larger the value, the greater the amount of image information and the more complex the image. The parameter value of the progress group is greater than that of the non-progress group (Figures 2, 3).

**Table 2:** Comparison of the selected radiomic features.

**Feature extraction methods**	**Radiomic features**	**Non-PD group (*n* = 39)**	**PD group (*n* = 33)**	***p*-value**
Fisher	Kurtosis	2.12 ± 3.92	0.78 ± 1.10	0.047
	“S(4,4)SumEntrp”	1.15 ± 0.17	1.21 ± 0.13	0.102^*^
	“S(5,0)AngScMom”	0.03 ± 0.02	0.02 ± 0.01	0.107^*^
	“S(5,5)SumEntrp”	1.14 ± 0.17	1.20 ± 0.13	0.108^*^
	“S(4,0)AngScMom”	0.03 ± 0.02	0.02 ± 0.01	0.110^*^
	“S(3,3)SumEntrp”	1.17 ± 0.16	1.22 ± 0.13	0.114^*^
	WavEnHH_s-5	97.44 ± 60.08	125.76 ± 92.12	0.122^*^
	“S(3,0)AngScMom”	0.03 ± 0.02	0.03 ± 0.01	0.123^*^
	“S(5,0)AngScMom”	0.03 ± 0.02	0.02 ± 0.01	0.165^*^
	“S(2,0)AngScMom”	0.04 ± 0.02	0.03 ± 0.01	0.180^*^
MI	“S(1,0)AngScMom”	0.04 ± 0.02	0.04 ± 0.01	0.140^*^
	“S(2,0)AngScMom”	0.03 ± 0.02	0.03 ± 0.01	0.236^*^
	“S(1,1)AngScMom”	0.04 ± 0.03	0.04 ± 0.02	0.398^*^
	“S(0,1)AngScMom”	0.05 ± 0.03	0.05 ± 0.02	0.175^*^
	“S(2,2)SumEntrp”	1.14 ± 0.31	1.24 ± 0.12	0.036^*^
	“S(1,0)SumEntrp”	1.18 ± 0.27	1.28 ± 0.11	0.046^*^
	“S(5,0)SumEntrp”	1.14 ± 0.17	1.19 ± 0.13	0.158^*^
	“S(2,0)AngScMom”	0.04 ± 0.02	0.03 ± 0.01	0.130^*^
	“S(5,5)Entropy”	1.81 ± 0.28	1.88 ± 0.21	0.235^*^
	“S(1,1)Entropy”	1.57 ± 0.25	1.63 ± 0.19	0.291^*^
POE + ACC	WavEnHH_s-4	63.58 ± 53.91	96.69 ± 145.33	0.191^*^
	Teta3	0.73 ± 0.15	0.76 ± 0.15	0.361^*^
	Kurtosis	2.12 ± 3.92	0.78 ± 1.10	0.047
	“S(5,0)SumAverg”	66.74 ± 4.91	68.13 ± 5.62	0.267^*^
	Teta1	0.90 ± 0.05	0.89 ± 0.04	0.512^*^
	WavEnLH_s-5	447.40 ± 354.50	371.19 ± 363.24	0.372^*^
	“S(4,4)SumVarnc”	18.34 ± 18.02	28.36 ± 53.11	0.272^*^
	“S(5,5)AngScMom”	0.03 ± 0.02	0.02 ± 0.01	0.177
	WavEnHH_s-2	2.99 ± 3.53	2.45 ± 1.64	0.424^*^
	“S(0,2)AngScMom”	0.04 ± 0.02	0.03 ± 0.01	0.172^*^

* *p-value is obtained by the t-test (normally distributed data); otherwise, p-value is obtained by the non-parametric test method Mann–Whitney U-test (non-normally distributed data)*.

*MI, mutual information; POE + ACC, classification error probability combined average correlation coefficients; Non-PD group, non-progressive group; PD group, progressive group*.

### Evaluation of the Value of Immunotherapy Through Three Classification Methods

**Figure 4:**
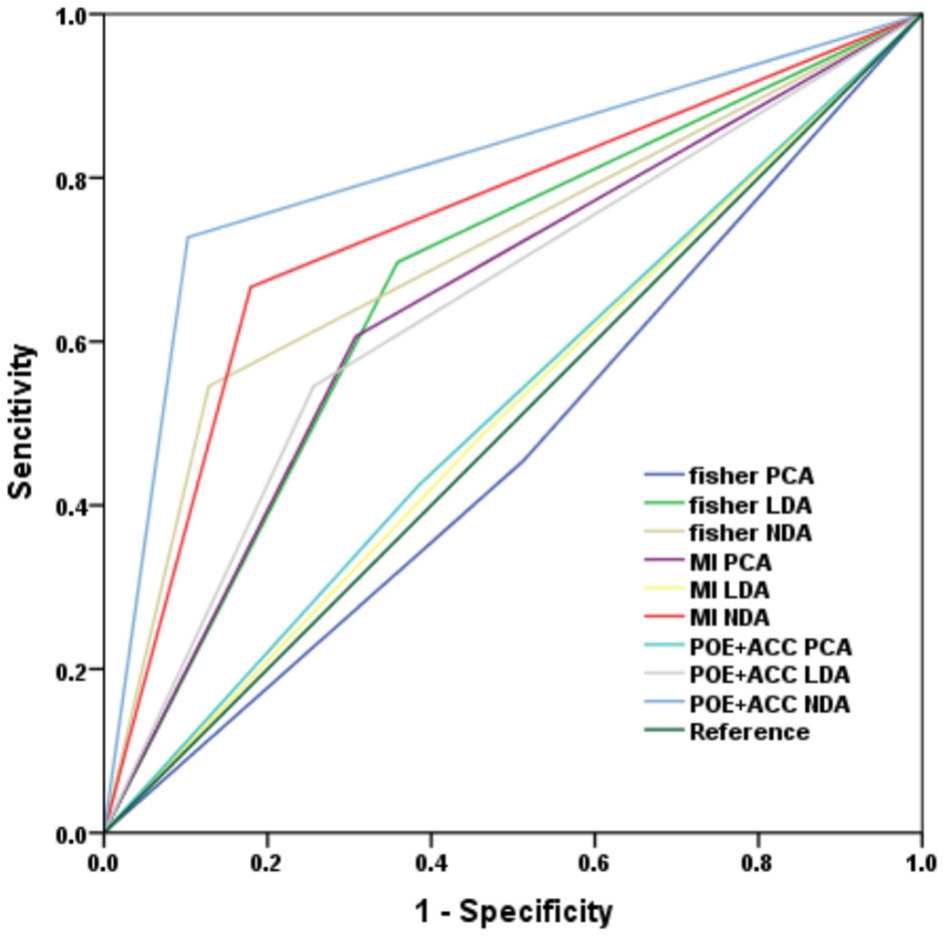
ROC curve of the three classification subtypes under each classifier model.

The three sets of texture features extracted by Fisher coefficient, MI, and POE + ACC methods are classified by PCA, LDA, and NDA methods, respectively (Table 3). The diagnostic efficacy of each method was further evaluated by receiver operating characteristic (ROC) analyses and calculated AUCs (Figure 4). The accuracy of various methods for predicting the therapeutic effect varies from 47.2 to 81.9%. The texture features extracted by the POE + ACC method have the best diagnostic efficacy by using the NDA classification method to predict the therapeutic effect (AUC = 0.812, 95% CI: 0.706–0.919). To predict the first effect after treatment, the sensitivity was 88.2%, the specificity was 76.3%, the accuracy was 81.9%, the positive predictive value was 76.9%, and the negative predictive value was 87.9%.

**Table 3:** Comparison of the performance metrics of the three classifiers.

**Feature extraction methods**	**Classifiers**	**AUC**	**95% CI**	***p***	**Sensitivity**	**Specificity**	**Accuracy**	**PPV**	**NPV**
Fisher	PCA	0.471	0.336,0.605	0.672	50%	40.6%	47.2%	51.3%	39.4%
	LDA	0.669	0.542,0.796	0.014	67.4%	65.5%	66.7%	74.4%	57.6%
	NDA	0.709	0.585,0.833	0.002	82.8%	65.1%	72.2%	61.5%	84.8%
MI	PCA	0.649	0.520,0.778	0.030	68.4%	61.7%	65.3%	66.7%	63.6%
	LDA	0.512	0.377,0.646	0.865	55%	46.9%	51.4%	56.4%	45.5%
	NDA	0.744	0.626,0.862	<0.001	80%	70.3%	75%	71.8%	78.8%
POE + ACC	PCA	0.520	0.385,0.655	0.773	57.1%	48.6%	52.8%	51.3%	54.5%
	LDA	0.645	0.515,0.774	0.036	70.6%	61.5%	65.3%	61.5%	69.7%
	NDA	0.812	0.706,0.919	<0.001	88.2%	76.3%	81.9%	76.9%	87.9%

*MI, mutual information; POE + ACC, classification error probability combined average correlation coefficients; PCA, principal component analysis; LDA, linear discriminant analysis; NDA, non-linear discriminant analysis; AUC, area under curve; CI, confidence interval; PPV, positive predictive value; NPV, negative predictive value*.

## Discussion

Modern immunotherapies play an important role in personalized cancer treatment. In oncological image monitoring, high-resolution CT is the standard for staging of the chest. However, special clinical manifestations such as pseudoprogression (PsPD), delayed response, and hyper-progressive disease (HPD) caused by infiltration of inflammatory cells and necrosis/edema of tumor tissue present a challenge (10). When clinicians confronted with atypical response patterns, it is difficult to evaluate the response and survival benefits. Therefore, they might be in a dilemma whether to continue immunotherapy or not. Thus, it is important to find robust non-invasive biomarkers on the basis of imaging that could allow prediction of patient response to immunotherapy and prognosis. The main research directions are functional and molecular imaging techniques, radiomics, and radiogenomics and the development of imaging biomarkers for immunotherapy (11). Our study used image texture analysis to analyze the texture features based on the contrast-enhanced CT images of tumor lesions before treatment. We extracted and classified features and predicted the efficacy of NSCLC immunotherapy according to the radiomics features. The 10 image texture features extracted by the POE + ACC method predicted the sensitivity of the tumor progression after treatment to be 88.2%, the specificity was 76.3%, and the accuracy rate was 81.9%. This result indicated that the radiomic signature can perceive the differences in the tumor microenvironment before treatment and provides valuable information for predicting the efficacy of immunotherapy.

Although solid tumor immunotherapy is currently widely practiced and has achieved some exciting results, there are still many unresolved problems. For example, immunotherapy lacks effective methods to find individualized tumor-specific targets (5); T lymphocytes, the main force of immunotherapy, generally have the disadvantages of decreased vitality, immune tolerance, and exhaustion of functions (12); immune cells cannot effectively penetrate infiltrating tumor tissues due to defects in their vascular structure and due to being rich in stroma (13); the tumor immunosuppressive microenvironment is intricate and monotherapy is not effective (14). Because the anti-tumor immune response is a complex process involving many immune cells and molecules, it is very complex and regulated by the body finely and dynamically. Therefore, compared with chemotherapy and targeted therapy, it is more challenging to find markers for predicting the efficacy of immunotherapy. At present, the commonly used efficacy prediction markers in clinical research of tumor immunotherapy include DNA mismatch repair defects, tumor cell PD-L1 overexpression, tumor mutation burden (TMB), etc. (15). In addition, different types of immune cells in the tumor microenvironment can also be used as markers for predicting the efficacy of immunotherapy. For example, CD8+ T cell infiltration often indicates a good response and prognosis for immunotherapy (16); a combination of different immune cells, such as CD3/CD8/CD45RO combined immune score (17), etc.

In recent years, with the development of medical image informatics, extraction of image features from medical images and analysis of clinical information have gradually attracted the attention of medical experts. In the field of oncology radiomics, breakthroughs have been made in the areas of differential diagnosis, pathological typing, metastasis assessment, and gene mutation prediction, especially for predicting the efficacy and prognosis (18). It has potentially great value for guiding and optimizing clinical decision-making as well as achieving individualized and precise treatment of lung cancer. A recent multi-cohort retrospective study published in the journal *Lancet Oncol*. also showed that the tumor infiltration CD8+ T cell imaging histology label can be used as an effective imaging biomarker for identifying tumor immunophenotypes and predicting PD-1/PD-L1 monoclonal antibody treatment efficacy (19). Vaidya et al. (20) and Tunali et al. (21) focused on hyper-progression of NSCLC, which not only segmented intratumor area but also delineated peritumoral region. Trebeschi et al. (22) used enhanced CT images before treatment to analyze the efficacy of anti-PD1 treatment in patients with melanoma and NSCLC by artificial intelligence (AI) technology. Moreover, genomics set analysis revealed some biological basis of the proposed biomarkers, which might be evident based on oncological decision-making. In our study, by comparing the texture features of contrast-enhanced CT images before treatment, the progressive group had larger S(2,2)SumEntrp and S(1,0)SumEntrp than the non-progressive group. Kurtosis values are smaller in the progressive group than in the non-progressive group. These texture features reflect that the lesions have large CT values and complex internal structure. The possible pathological mechanism that these characteristics affect the efficacy of immunotherapy is that defect of the tumor tissue vascular structure and rich stroma make it difficult for immune cells to penetrate effectively and infiltrate; the tumor immunosuppressive microenvironment is complicated, and the monotherapy is not effective (23, 24). This result coincides with the reason why we chose the enhanced image for analysis, that the immune status of the tumor is substantially influenced by its degree of vascularization (25).

Our study has some limitations. First, the sample size is small and comes from a single center. We will continue to expand the sample size, including multi-center data to further verify the reliability of the conclusion. Second, the image texture analysis in this study is based on 2D images (central cross-sectional images of target lesions) to represent the entire lesion, and results may be biased. In the next study, we will use 3D images to extract the entire tumor to minimize the bias caused by this factor.

## Conclusions

In short, through texture analysis of the baseline contrast-enhanced chest CT imaging before treatment and texture feature extraction, the efficacy prediction of NSCLC immunotherapy can be achieved. The highest prediction efficiency is sensitivity, specificity, and accuracy rate were 88.2%, 76.3%, and 81.9%, respectively. Radiomics texture provides a new method for early clinical evaluation of the NSCLC immunotherapy efficacy prediction.

## Data Availability Statement

The raw data supporting the conclusions of this article will be made available by the authors, without undue reservation.

## Ethics Statement

The studies involving human participants were reviewed and approved by Institutional Ethics Review Committee of the Shanghai Chest Hospital. Written informed consent for participation was not required for this study in accordance with the national legislation and the institutional requirements.

## Author Contributions

The literature search, analysis, data explanation, and manuscript draft were finished by LS. LS, HF, and XY are responsible for the analysis and explanation of the radiomics imaging features data. HF, GT, and XL acquired the clinical information and imaging. ZY and XY designed the study, explained the data, and made multiple revisions to the manuscript. All authors contributed to the article and approved the submitted version.

## Conflict of Interest

The authors declare that the research was conducted in the absence of any commercial or financial relationships that could be construed as a potential conflict of interest.
